# Dual immunomodulator therapy with adalimumab and upadacitinib to treat recalcitrant hidradenitis suppurativa

**DOI:** 10.1016/j.jdcr.2024.12.008

**Published:** 2024-12-16

**Authors:** Zahidul Islam, Solbie Choi, Lucy Wang, Tyler M. Andriano, Kristina Campton

**Affiliations:** a Division of Dermatology, Albert Einstein College of Medicine, Bronx, New York; b School of Medicine, New York Medical College, Valhalla, New York

**Keywords:** double-dose adalimumab, dual immunomodulator therapy, hidradenitis suppurativa, JAK and anti-TNF therapy, Janus kinase (JAK) inhibitors, refractory, TNF- α inhibitor, upadacitinib

## Introduction

Hidradenitis suppurativa (HS) is a chronic inflammatory skin condition of the hair follicles characterized by painful, purulent nodules, and draining tunnels, primarily occurring in intertriginous regions, but can occur in other areas of the body. Current treatments focus on a multifactorial approach to target the inflammatory, hormonal, microbial, mechanical, and lifestyle factors involved in HS.^1^ The inflammatory response of HS is believed to involve increased proinflammatory cytokines including tumor necrosis factor-alpha, interferon-gamma, and interleukins (ILs). Consequently, there has been a growing body of research on biologics targeting these cytokines; adalimumab and secukinumab, an injectable monoclonal antibody to inhibit tumor necrosis factor-alpha and IL-17, respectively, have been approved for treating HS.

Furthermore, dysregulation of the Janus kinase/signal transducers and activators of transcription (JAK/STAT) signaling pathway, specifically tumor necrosis factor-alpha and interferon-gamma induced JAK/STAT1 signaling, has been found to trigger HS, making JAK inhibitors a promising therapeutic target.^2^ Upadacitinib is a selective JAK1 inhibitor that has shown initial promise in moderate to severe HS, although further studies are needed to confirm its safety and efficacy.^3^ The inflammatory pathogenesis of HS suggests a potential benefit in the combination of biologics and JAK inhibitors. Here, we present a unique case in treating refractory HS with concomitant adalimumab and upadacitinib therapy.

## Case presentation

A 25-year-old male, nonsmoker, nonobese with a history of acne and atopic dermatitis, presented with a 6-year history of HS. In 2021, he was prescribed chlorhexidine wash, clindamycin gel, oral doxycycline, trimethoprim-sulfamethoxazole, finasteride, and infliximab 7.5 mg/kg every 4 weeks. He was then switched from infliximab to adalimumab due to the birth of his newborn, the inconvenience of infusions, and the higher co-pay. 80 mg of adalimumab weekly was initiated toward the end of 2022, as he previously failed the standard bi-weekly 80 mg dosing. Improvement in the frequency and pain of his flares on this regimen lasted for about 10 months. He then developed a recurrence of his severely painful flares with significant drainage, which left him debilitated and unable to perform his job and household tasks.

**Fig 1:**
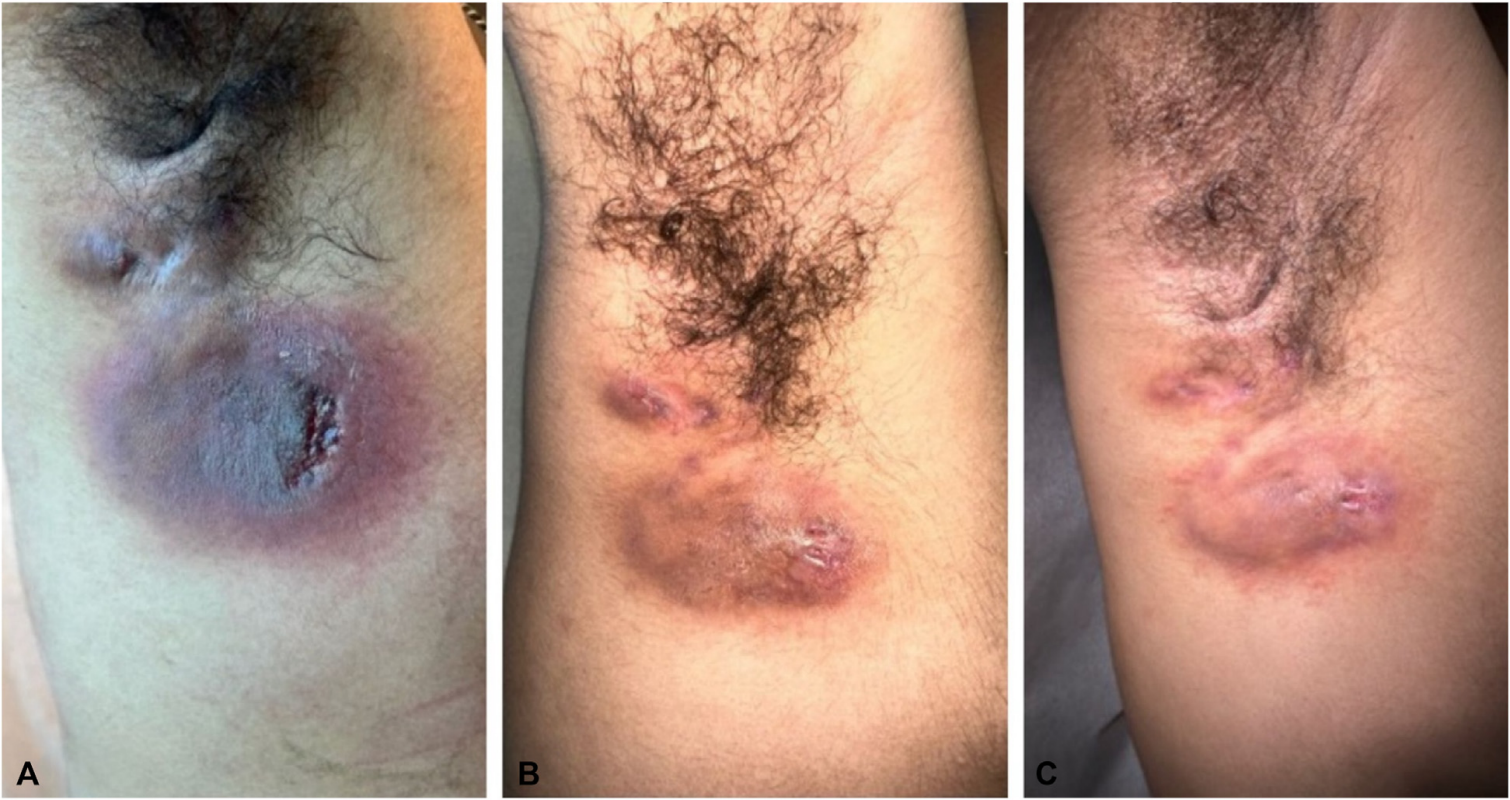
Right axilla lesion. Improvement of erythema, size, and drainage of right axilla abscess/tunnel from initial visit (**A**), at a 2-month follow-up visit (**B**), and to a 4-month follow-up visit (**C**).

His HS symptoms continued to worsen as he experienced abscesses and inflamed nodules on both axillae, inguinal folds, and intergluteal areas with an overall International HS Severity Score System (IHS4) of 13. Due to an increased frequency of flares, failure of multiple therapies, and the patient’s inability to attend infusions, upadacitinib 30 mg daily was added to his regimen of topical antimicrobials, trimethoprim-sulfamethoxazole, finasteride, and adalimumab. At a 2-month follow-up, he displayed significant improvement, with decreased drainage, size, and inflammation of previous lesions (Fig 1). His complete blood count and liver tests were normal. His IHS4 was an 8 and he reported no side effects with the combination of upadacitinib and adalimumab. He was discontinued on adalimumab and continued on upadacitinib 45 mg daily. At 4 months, he continued to show improvement and experienced less frequent flares. On physical exam, the size, erythema, draining, and tenderness of the abscess of his right axilla were decreased and the nodules on his left axilla, left inguinal fold, and pubis flattened. His IHS4 was a 4 and he continued to report no adverse effects.

## Discussion

The approved dosage for adalimumab for HS is either 40 mg weekly or 80 mg bi-weekly.^4^ However, our patient did not respond adequately to standard dosage and was subsequently increased to 80 mg weekly. This dosing is in line with a case series of 14 HS patients on 80 mg of adalimumab weekly, who reported significant improvements in their IHS4, pain index, HS-Physician Global Assessment, and Cardiff Dermatology Life Quality Index.^5^

Despite the initial improvements with adalimumab, our patient developed a tolerance to the medication after 10 months as he reported a plateau in symptom improvement and relapse of his painful flares. Given his previous failed response to standard HS treatments and his inability to attend infliximab infusions, the decision was made to incorporate upadicitinib 30 mg into his current regimen. Although studies on the use of upadacitinib for HS are limited, phase 2 trials demonstrated that 20 HS patients experienced an improved clinical response to this dose and proteomic analysis identified a significant decrease in signaling molecules that are linked with the inflammatory pathways of HS.^6^^,^^7^

In response to inflammation, macrophages release TNF-alpha, which then binds to respective receptors and activates nuclear factor-kappa B and mitogen-activated protein kinases to promote gene expression that involves immune response and inflammation. Adalimumab is a human, recombinant monoclonal TNF-alpha inhibitor that exerts its effect via distorting cytokine-driven inflammation.^8^ Upadacitinib is a second-generation JAK1 inhibitor that blocks the production of pro-inflammatory cytokines and growth factors such as interleukin IL-2, IL-4, IL-6, IL-23, and interferon-gamma.^9^ The cytokines that JAK1 inhibitors target, prevent T-cell activation, differentiation, and proliferation. While these 2 medications affect different immunologic pathways, they have both been approved for treating chronic autoimmune conditions such as rheumatoid arthritis, psoriatic arthritis, atopic dermatitis, ankylosing spondylitis, Crohn's disease, and ulcerative colitis which motivated us to explore their effects on another chronic autoimmune condition.^8^^,^^9^ Although there is no published literature on the use of combination therapy with these two drugs, we postulated that our patient would benefit from inhibition of two inflammatory pathways associated with HS, because minimal improvement was seen targeting a single pathway.

Though this case demonstrates promise for combination biologic therapy, it is important to note that the use of upadacitinib alone may have promoted an improvement in this patient, rather than the combination of upadacitinib and adalimumab. In this instance, upadacitinib was able to be continued as monotherapy as it also addressed the patient's concomitant atopic dermatitis. Despite efficacy, clinicians should be cautious and monitor for tuberculosis and other infections as well as heart disease, liver function, signs of cancer, anemias, and allergic reactions as they may be compounded by dual biologic and JAK therapy. With its complex etiology, HS often requires patients to be on multiple drugs that target antibiotics, hormones, and biologics. Our study presents a new combination of drugs that can improve severe HS symptoms. Although it is limited to a single case, further studies are needed to evaluate the efficacy of biologic and JAK monotherapies versus combination therapies.

## Conflicts of interest

None disclosed.
